# ﻿Four new species of *Phanerochaete* (Polyporales, Basidiomycota) from China

**DOI:** 10.3897/mycokeys.111.133093

**Published:** 2024-12-03

**Authors:** Kai-Yue Luo, Xin Zhang, Yu-Cheng Dai, Yuan Yuan

**Affiliations:** 1 State Key Laboratory of Efficient Production of Forest Resources, School of Ecology and Nature Conservation, Beijing Forestry University, Beijing 100083, China Beijing Forestry University Beijing China

**Keywords:** Molecular phylogeneny, Polyporales, taxonomy, wood-decaying fungi, white rot

## Abstract

Four new wood-inhabiting fungi viz. *Phanerochaetecastanea*, *P.citrinoalba*, *P.citrinorhizomorpha*, and *P.wuyiensis***spp. nov.** – are proposed based on a combination of morphological features and molecular evidence. *Phanerochaetecastanea* is characterized by soft coriaceous basidiomata detachable from the substrate, becoming reddish brown in KOH, subulate cystidia with an obtuse apex. *Phanerochaetecitrinoalba* is characterized by the coriaceous basidiomata with smooth, cracking hymenial surface, sterile margins with yellowish to whitish rhizomorphs, a monomitic hyphal system, generative hyphae mostly with simple septa and occasionally with clamp connections at basal hyphae. *Phanerochaetecitrinorhizomorpha* is characterized by soft coriaceous basidiomata with a salmon to peach hymenial surface, a sterile margin with yellowish rhizomorphs, simple septate generative hyphae, and clavate to subfusiform or subulate cystidia with an obtuse apex. *Phanerochaetewuyiensis* is characterized by membranaceous basidiomata with smooth or locally tuberculate hymenial surface and the whitish rhizomorphs, generative hyphae with both simple septa and clamp connections at basal hyphae, cystidia projecting above hymenium. DNA sequences of the ITS and LSU markers of the studied samples were generated, and phylogenetic analyses were performed with Maximum Likelihood and Bayesian Inference methods. The phylogenetic tree inferred from the concatenated ITS+nLSU dataset highlighted the placement of the four new species in the genus *Phanerochaete* (Phanerochaetaceae, Polyporales). Phylogenetically related and morphologically similar species to these four new species are discussed. Furthermore, an identification key to accepted species of *Phanerochaete* in China is given.

## ﻿Introduction

The taxa in the family Phanerochaetaceae are mostly corticioid fungi, especially *Phanerochaete*, as a major member of the family. It is a large genus with diverse morphological features, and it is widely distributed from boreal to tropical forests. It causes a white rot on all kinds of wood and plays an important role in carbon cycling (Burdsall 1985; Larsson et al. 2004; Bernicchia and Gorjón 2010; Ryvarden and Melo 2014; Ghobad-Nejhad et al. 2015; Nagy et al. 2017; Wu 2000; Xu et al. 2020; Yuan et al. 2023).

*Phanerochaete* P. Karst. is typified by *P.alnea* (Fr.) P. Karst. and has a worldwide distribution (Spirin et al. 2017). It is characterized by resupinate, membranaceous basidiomata, smooth or tuberculate hymenial surface, a monomitic hyphal system, generative hyphae mostly simple septate, the presence of smooth or encrusted cystidia, thin-walled, non-amyloid, and acyanophilous basidiospores, and causing a white-rot (Wu 2000; Wu et al. 2010; Floudas and Hibbett 2015; Ghobad-Nejhad et al. 2015). Currently, MycoBank (https://www.mycobank.org/page/Simple%20names%20search) and Index Fungorum (https://www.indexfungorum.org/Names/Names.asp?pg=1) have registered 214 records and 202 records in *Phanerochaete*, respectively. About 100 species are currently accepted in *Phanerochaete* worldwide, of which 51 have been found in China (including the four new species presented here) as of June 2024 (Xu et al. 2020; Boonmee et al. 2021; Chen et al. 2021; Wang and Zhao 2021; Li et al. 2023; Yu et al. 2023; Zhang et al. 2023; Deng et al. 2024).

Taxonomists used the membranaceous nature of the basidiomata, the monomitic hyphal system, the presence of clamp connections (simple, double or multiple clamps per septum) or simple septa, and the presence of cystidia as characters for the delimitation of the genus. The simplicity of the morphological features characterise *Phanerochaete* and the existence of species with basidiomata that fulfil only some of these morphological criteria render the limits of the genus uncertain (Floudas and Hibbett 2015).

Recent phylogenetic studies show that *Phanerochaete* s.l. is polyphyletic, and several new genera have been introduced, some of them placed into different families, or even orders (Greslebin et al. 2004; Wu et al. 2010; Binder et al. 2013; Floudas and Hibbett 2015). Most *Phanerochaete* species are still retained in a monophyletic lineage (Phanerochaetaceae sensu Larsson 2007) within Polyporales, along with genera such as *Hyphodermella*, *Phlebiopsis*, and *Rhizochaete* (Larsson 2007; Wu et al. 2010; Floudas and Hibbett 2015; Miettinen et al. 2016). Most members of the genus are nested in the phlebioid clade, comprising a number of *Phanerochaete* species assembled in a highly supported clade, referred to as the core *Phanerochaete* clade (Wu et al. 2010; Floudas and Hibbett 2015; Justo et al. 2017; Chen et al. 2021).

During investigations on the wood-inhabiting fungi in the Xizang Autonomous Region, Zhejiang, and Yunnan Province of China, samples corresponding to *Phanerochaete* were collected, and four species were initially identified as potentially new by morphology. To clarify the placement and relationships of the four species, we carried out a phylogenetic and morphological studies on *Phanerochaete* in China.

## ﻿Materials and methods

### ﻿Morphological studies

The studied specimens were collected from wild forests and are deposited in the Fungarium of the Institute of Microbiology, Beijing Forestry University (BJFC). Morphological descriptions are based on field notes and voucher specimens. The microscopic analysis follows Spirin et al. (2017). Freehand sections were made from dried basidiomata and mounted in 2% (w/v) potassium hydroxide (KOH) to observe color changes. Sections were studied at a magnification of up to 1000× using a Nikon Eclipse 80i microscope and phase contrast illumination. Microscopic features and measurements were made from slide preparations stained with Cotton Blue and Melzer’s reagent. To represent the variation in the size of spores, 5% of measurements were excluded from each end of the range and are given in parentheses. In the description: **KOH** = 5% potassium hydroxide, **IKI** = Melzer’s reagent, **IKI+** = amyloid or dextrinoid, **IKI-** = neither amyloid nor dextrinoid, **CB** = Cotton Blue, **CB+** = cyanophilous in Cotton Blue, **CB-** = acyanophilous in Cotton Blue, **L** = arithmetic average of spore length, **W** = arithmetic average of spore width, **Q** = L/W ratios and **n** = number of basidiospores measured from given number of specimens. Colour terms follow Anonymous (1969) and Petersen (1996).

### ﻿DNA extraction, amplification, and sequencing

A CTAB rapid plant genome extraction kit-DN14 (Aidlab Biotechnologies Co., Ltd, Beijing) was used to obtain DNA from dried specimens and to perform the polymerase chain reaction (PCR) according to the manufacturer’s instructions with some modifications (Shen et al. 2019; Sun et al. 2020). The internal transcribed spacer (ITS) and large subunit nuclear ribosomal RNA gene (nLSU) were amplified using the primer pairs ITS5/ITS4 and LR0R/LR7 (White et al. 1990; Hopple and Vilgalys 1999) (https://sites.duke.edu/vilgalyslab/rdna_primers_for_fungi/).

The PCR procedure for ITS was as follows: initial denaturation at 95 °C for 3 min, followed by 34 cycles at 94 °C for 40 s, annealing at 54 °C for 45 s and extension at 72 °C for 1 min, and a final extension of 72 °C for 10 min. The PCR procedure for nLSU was as follows: initial denaturation at 94 °C for 1 min, followed by 34 cycles of denaturation at 94 °C for 30 s, annealing at 50 °C for 1 min, and extension at 72 °C for 1.5 min, and a final extension at 72 °C for 10 min. The PCR products were purified and sequenced at the Beijing Genomics Institute (BGI), China, with the same primers. DNA sequencing was performed at the Beijing Genomics Institute and the newly generated sequences were deposited in GenBank (Sayers et al. 2024). All sequences analysed in this study are listed in Table 1. Sequences generated from this study were aligned manually with additional sequences downloaded from GenBank using AliView version 1.27 (Larsson 2014). The final ITS and nLSU datasets were subsequently aligned using MAFFT v.7 under the E-INS-i strategy with no cost for opening gaps and equal cost for transformations (command line: mafft –genafpair –maxiterate 1000) (Katoh and Standley 2013) and visualised in AliView. Alignments were spliced and transformed formats in Mesquite v.3.2. (Maddison and Maddison 2017). Multiple sequence alignments were trimmed by trimAI v.1.2 using the -htmlout-gt 0.8 -st option to deal with gaps, when necessary (Capella-Gutierrez et al. 2009).

**Table 1.** Names, specimen numbers, references, and corresponding GenBank accession numbers of the taxa used in the phylogenetic analysis of this study. [New species are shown in bold, * type material; type specimens of other species are shown in bold].

Species name	Specimen No.	GenBank accession No.		Country	References
		ITS	LSU		
*Phanerochaeteaculeata*	Wu 1809278	MZ422786	MZ637178	China	Chen et al. (2021)
*P.aculeata*	GC 1703117	MZ422785	MZ637177	China	Chen et al. (2021)
*P.albida*	WEI 18365	MZ422789	MZ637180	China	Chen et al. (2021)
*P.albida*	GC 140714	MZ422788	MZ637179	China	Chen et al. (2021)
*P.alnea*	K. H. Larsson 12054	KX538924	—	Norway	Spirin et al. (2017)
*P.alpina*	Wu 130861	MZ422790	MZ637182	China	Chen et al. (2021)
*P.alpina*	Wu 130877	MZ422791	MZ637183	China	Chen et al. (2021)
***P.arizonica***	**RLG 10248**	**KP135170**	**KP135239**	**USA**	**Floudas and Hibbett (2015)**
*P.australis*	He 6013	MT235656	MT248136	China	Xu et al. (2020)
*P.australis*	HHB 7105	KP135081	KP135240	USA	Floudas and Hibbett (2015)
*P.australosanguinea*	MA Fungi 91308	MH233925	MH233928	Chile	Phookamsak et al. (2019)
***P.australosanguinea***	**MA Fungi 91309**	**MH233926**	**MH233929**	**Chile**	**Phookamsak et al. (2019)**
*P.bambusicola*	He 3606	MT235657	MT248137	China	Chen et al. (2021)
***P.bambusicola***	**Wu 0707-2**	**MF399404**	**MF399395**	**China**	**Xu et al. (2020)**
*P.brunnea*	He 4192	MT235658	MT248138	China	Phookamsak et al. (2019)
*P.burdsallii*	RF9JR	KU668973	—	USA	Yu et al. (2023)
***P.burdsallii***	**He 2066**	**MT235690**	**MT248177**	**USA**	**Xu et al. (2020)**
*P.burtii*	HHB 4618	KP135117	KP135241	USA	Floudas and Hibbett (2015)
*P.burtii*	FD 171	KP135116	—	USA	Floudas and Hibbett (2015)
*P.calotricha*	Vanhanen 382	KP135107	—	USA	Floudas and Hibbett (2015)
*P.canobrunnea*	He 5726	MT235659	MT248139	Sri Lanka	Phookamsak et al. (2019)
*P.canobrunnea*	CHWC 150666	LC412095	LC412104	China	Phookamsak et al. (2019)
*P.canolutea*	LWZ 202109214a	ON897909	ON885366	China	Unpublished
*P.canolutea*	TNM F14823	NR175166	NG153829	China	Chen et al. (2021)
*P.carnosa*	He 5172	MT235660	MT248140	China	Phookamsak et al. (2019)
*P.carnosa*	HHB 9195	KP135129	KP135242	USA	Floudas and Hibbett (2015)
***P.castanea***	Dai 24915*	**PP960566**	**PP960569**	**China**	**Present study**
***P.castanea***	**Dai 24916**	**PP960567**	**PP960570**	**China**	**Present study**
*P.chrysosporium*	He 5778	MT235661	MT248141	Sri Lanka	Phookamsak et al. (2019)
***P.chrysosporium***	**HHB 6251**	**KP135094**	**KP135246**	**USA**	**Floudas and Hibbett (2015)**
***P.citrinoalba***	Dai 26584*	**PP779892**	**PP779887**	**China**	**Present study**
***P.citrinorhizomorpha***	**Dai 20753**	**PP960568**	**PP960571**	**China**	**Present study**
***P.citrinorhizomorpha***	Dai 26417*	**PP779891**	**PP779886**	**China**	**Present study**
*P.citrinosanguinea*	FP 105385	KP135100	KP135234	USA	Floudas and Hibbett (2015)
*P.concrescens*	He 4657	MT235662	MT248142	China	Chen et al. (2021)
***P.concrescens***	**H Spirin 7322**	**KP994380**	**KP994382**	**Russia**	**Volobuev et al. (2015)**
*P.conifericola*	OM 8110	KP135171	—	Finland	Floudas and Hibbett (2015)
*P.crystallina*	Chen 3823	MZ422802	MZ637188	China	Chen et al. (2021)
***P.crystallina***	**Chen 3576**	**MZ422801**	—	**China**	**Chen et al. (2021)**
*P.cumulodentata*	He 2995	MT235664	MT248144	China	Xu et al. (2020)
*P.cumulodentata*	LERUS 298935	KP994359	KP994386	Russia	Volobuev et al. (2015)
*P.cystidiata*	He 4224	MT235665	MT248145	China	Phookamsak et al. (2019)
*P.cystidiata*	Wu 1708-326	LC412097	LC412100	China	Wu et al. (2018)
*P.ericina*	HHB 2288	KP135167	KP135247	USA	Floudas and Hibbett (2015)
*P.ericina*	He 4285	MT235666	MT248146	China	Phookamsak et al. (2019)
*P.fusca*	Wu 1409-163	LC412099	LC412106	China	Wu et al. (2018)
***P.guangdongensis***	**Wu 1809-348**	**MZ422813**	**MZ637199**	**China**	**Chen et al. (2021)**
*P.guangdongensis*	Wu 1809-319	MZ422811	MZ637197	China	Chen et al. (2021)
***P.hainanensis***	**He 3562**	**MT235692**	**MT248179**	**China**	**Boonmee et al. (2021)**
*P.incarnata*	He 201207281	MT235669	MT248149	China	Phookamsak et al. (2019)
*P.incarnata*	WEI 16075	MF399406	MF399397	China	Xu et al. (2020)
*P.krikophora*	HHB 5796	KP135164	KP135268	USA	Floudas and Hibbett (2015)
*P.laevis*	He 20120917-8	MT235670	MT248150	China	Phookamsak et al. (2019)
*P.laevis*	HHB 15519	KP135149	KP135249	USA	Floudas and Hibbett (2015)
***P.leptocystidiata***	**He 5853**	**MT235685**	**MT248168**	**China**	**Xu et al. (2020)**
*P.leptocystidiata*	Dai 10468	MT235684	MT248167	China	Xu et al. (2020)
*P.livescens*	He 5010	MT235671	MT248151	China	Phookamsak et al. (2019)
*P.magnoliae*	He 3321	MT235672	MT248152	China	Phookamsak et al. (2019)
*P.metuloidea*	He 2766	MT235682	MT248164	China	Phookamsak et al. (2019)
***P.minor***	**He 3988**	**MT235686**	**MT248170**	**China**	**Phookamsak et al. (2019)**
*P.parmastoi*	He 4570	MT235673	MT248153	China	Phookamsak et al. (2019)
*P.pruinosa*	CLZhao 7112	MZ435346	MZ435350	China	Wang and Zhao (2021)
***P.pruinosa***	**CLZhao 7113**	**MZ435347**	**MZ435351**	**China**	**Wang and Zhao (2021)**
*P.porostereoides*	He 1902	KX212217	KX212221	China	Liu and He (2016)
***P.pseudomagnoliae***	**PP 25**	**KP135091**	**KP135250**	**South Africa**	**Floudas and Hibbett (2015)**
***P.pseudosanguinea***	**FD 244**	**KP135098**	**KP135251**	**USA**	**Floudas and Hibbett (2015)**
***P.rhizomorpha***	**GC 1708-335**	**MZ422824**	**MZ637208**	**China**	**Chen et al. (2021)**
*P.rhizomorpha*	GC 1708-354	MZ422825	MZ637209	China	Chen et al. (2021)
*P.rhodella*	FD 18	KP135187	KP135258	USA	Floudas and Hibbett (2015)
*P.sanguineocarnosa*	FD-359	KP135122	KP135245	USA	Floudas and Hibbett (2015)
***P.sinensis***	**He 4660**	**MT235688**	**MT248175**	**China**	**Xu et al. (2020)**
*P.sinensis*	GC 180956	MT235689	MT248176	China	Xu et al. (2020)
*P.singularis*	He1873	KX212220	KX212224	China	Liu and He (2016)
***P.spadicea***	**Wu 0504-15**	**MZ422837**	**MZ637219**	**China**	**Chen et al. (2021)**
*P.spadicea*	Wu 0504-11	MZ422836	—	China	Chen et al. (2021)
*P.stereoides*	He 5824	MT235677	MT248158	Sri Lanka	Phookamsak et al. (2019)
*P.stereoides*	He 2309	KX212219	KX212223	China	Liu and He (2016)
***P.subcarnosa***	**Wu 9310-3**	**MZ422841**	**GQ470642**	**China**	**Wu et al. (2010)**
*P.subcarnosa*	GC 1809-90	MZ422840	MZ637222	China	Chen et al. (2021)
*P.subceracea*	HHB-9434	KP135163	—	USA	Floudas and Hibbett (2015)
***P.subrosea***	**He 2421**	**MT235687**	**MT248174**	**China**	**Phookamsak et al. (2019)**
*P.subsanguinea*	CLZhao 10470	MZ435348	MZ435352	China	Wang and Zhao (2021)
***P.subsanguinea***	**CLZhao 10477**	**MZ435349**	**MZ435353**	**China**	**Wang and Zhao (2021)**
*P.subtuberculata*	CLZhao F5130	OP605484	OQ195088	China	Yu et al. (2023)
***P.subtuberculata***	**CLZhao F6838**	**OP605485**	**OQ195087**	**China**	**Yu et al. (2023)**
*P.taiwaniana*	He 5269	MT235680	MT248161	VietNam	Phookamsak et al. (2019)
*P.taiwaniana*	Wu 011213	MF399412	MF399403	China	Xu et al. (2020)
*P.subtropica*	CLZhao F2763	OP605518	OQ195090	China	Yu et al. (2023)
***P.subtropica***	**CLZhao F8716**	**OP605486**	**OQ195089**	**China**	**Yu et al. (2023)**
*P.velutina*	He 3079	MT235681	MT248162	China	Phookamsak et al. (2019)
*P.velutina*	Kotiranta 25567	KP994354	KP994387	Russia	Volobuev et al. (2015)
***P.wuyiensis***	Dai 25530*	**PP779888**	**PP779883**	**China**	**Present study**
***P.wuyiensis***	**Dai 26246**	**PP779889**	**PP779884**	**China**	**Present study**
***P.wuyiensis***	**Dai 26250**	**PP779890**	**PP779885**	**China**	**Present study**
***P.yunnanensis***	**He 2719**	**MT235683**	**MT248166**	**China**	**Xu et al. (2020)**
*Riopametamorphosa*	Viacheslav Spirin 2395	KX752601	KX752601	Russia	Miettinen et al. (2016)
*R.pudens*	Otto Miettinen 8772	KX752598	—	USA	Miettinen et al. (2016)

### ﻿Phylogenetic analyses

The two-marker DNA multiple sequence alignment (ITS+nLSU) was used to determine the phylogenetic position of the new species. The multiple sequence alignments and the retrieved topologies were deposited in Figshare (https://figshare.com/) under accession DOI: 10.6084/m9.figshare.27683265. Sequences of *Riopametamorphosa* (Fuckel) Miettinen & Spirin and *R.pudens* Miettinen, obtained from GenBank, were used as the outgroups (Miettinen et al. 2016). The phylogenetic analyses followed the approach of Han et al. (2015) and Zhu et al. (2019). Maximum Likelihood (ML) and Bayesian Inference (BI) analyses were performed based on the ITS+nLSU datasets.

Sequences were analysed using Maximum Likelihood (ML) with RAxML-HPC2 through the CIPRES Science Gateway (www.phylo.org; Miller et al. 2010). Branch support (BT) for ML analysis was determined by 1,000 bootstrap replicates. Bayesian phylogenetic inference and Bayesian Posterior Probabilities (BPP) were computed with MrBayes 3.2.6 (Ronquist and Huelsenbeck 2003). Four Markov chains were run for 1.6 million generations (two-marker dataset) until the split deviation frequency value was less than 0.01 and trees were sampled every 100 generations. The first 25% of the sampled trees were discarded as burn-in and the remaining ones were used to reconstruct a majority rule consensus and calculate Bayesian Posterior Probabilities (BPP) of the clades. All trees were viewed in FigTree v. 1.4.3 (http://tree.bio.ed.ac.uk/software/figtree/). Branches that received bootstrap support for ML (≥ 75% (ML-BS)) and BPP (≥ 0.95 BPP) were considered as significantly supported. The ML bootstrap (ML) ≥ 50% and BBP (BPP) ≥ 0.90 are presented on topologies from ML analysis, respectively.

## ﻿Results

### ﻿Molecular phylogeny

**Figure 1.**
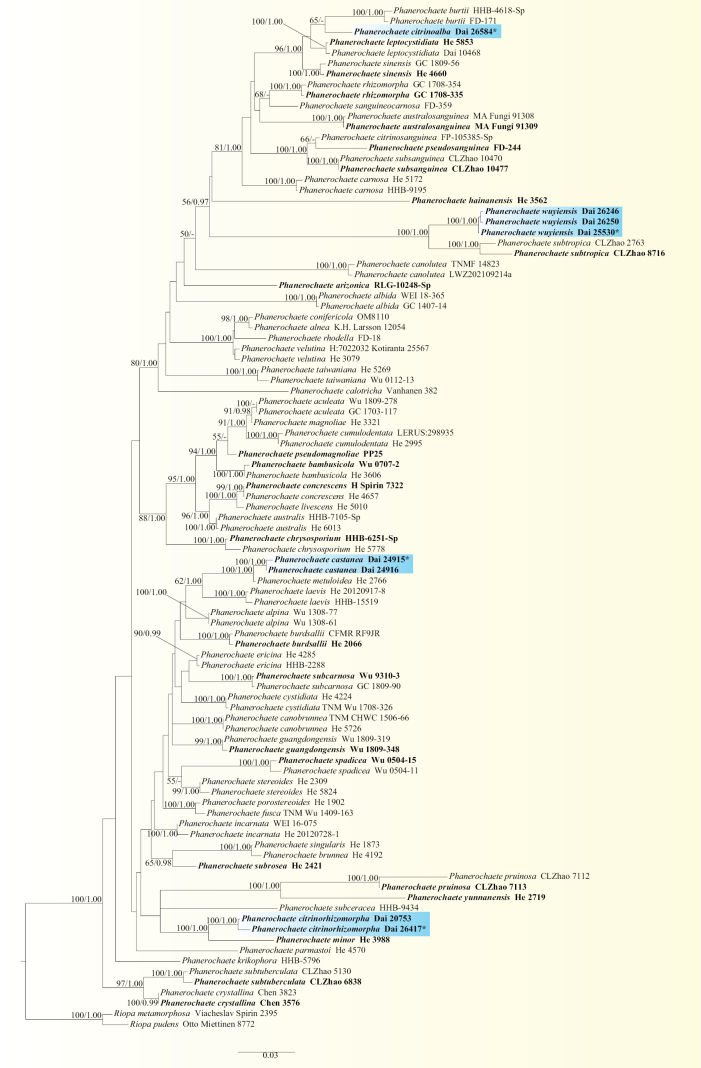
ML analysis of *Phanerochaete* based on dataset of ITS+nLSU. ML bootstrap values equal to or higher than 50% and Bayesian posterior probabilities values equal to or higher than 0.90 are shown. New taxa are in bold, * represents type material and in blue colour. Type specimens for all species are in bold.

The combined two-marker dataset (ITS+nLSU) included sequences from 97 samples representing 61 taxa. The phylogenetic reconstruction performed with Maximum Likelihood (ML) and Bayesian Inference (BI) analyses for the combined dataset showed similar topology and few differences in statistical support. The best model-fit applied in the Bayesian analysis was GTR+I+G, lset nst = 6, rates = invgamma and prset statefreqpr = dirichlet (1, 1, 1, 1). Bayesian analysis resulted in a nearly congruent topology with an average standard deviation of split frequencies = 0.014712 to ML analysis and thus, only the ML tree is shown (Fig. 1).

The phylogenetic tree inferred from the ITS+nLSU sequences indicated that the four new species belonged to *Phanerochaete* (Fig. 1). In addition, *Phanerochaetecastanea* grouped together with *P.metuloidea* Y.L. Xu & S.H. He with high support (ML = 100, BPP = 1.00); *P.citrinoalba* was sister to *P.burtii* (Romell ex Burt) Parmasto, with a low support (65% BS); *P.citrinorhizomorpha* grouped together with *P.minor* Y.L. Xu & S.H. He, with a high support (ML = 100, BPP = 1.00); and *P.wuyiensis* was sister to *P.subtropica* J. Yu & C.L. Zhao with high support (ML = 100, BPP = 1.00).

### ﻿Taxonomy

#### ﻿ Phanerochaete castanea

Taxon classificationFungiPolyporalesPhanerochaetaceae

K.Y. Luo, Yuan Yuan, Y.C. Dai & Xin Zhang sp. nov.

4D789954-CD4F-5BF3-BEB2-1B322FC4E966

854762

Figs 2 , 3

##### Holotype.

China • Zhejiang Province, Jinhua, Wuyi County, Niutoushan Forest Park; on a rotten bamboo; 17.VI.2023; Y.C. Dai 24915 (BJFC042468).

**Figure 2.**
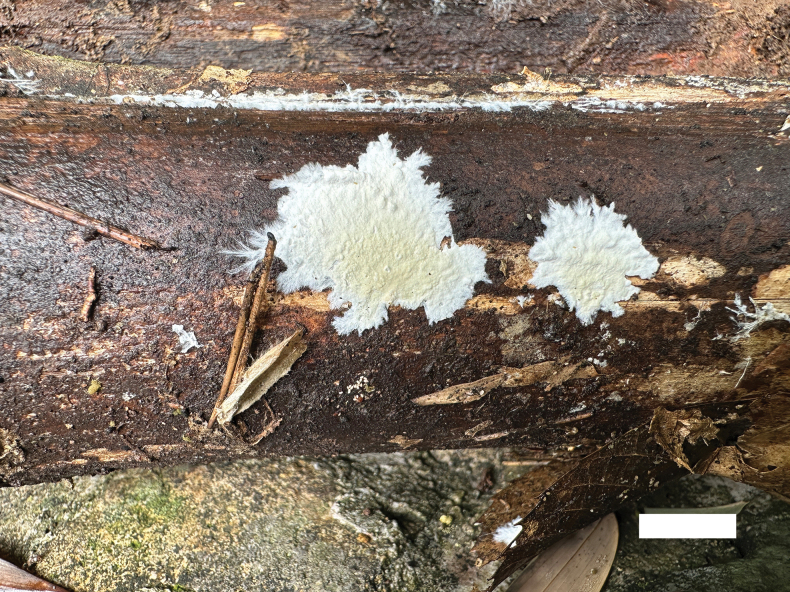
Basidiomata of *Phanerochaetecastanea* (Holotype, Dai 24915). Scale bar: 0.5 cm.

##### Etymology.

*Castanea* (Lat.) refers to the colour of new species’ basidiomata turning reddish brown in KOH.

**Figure 3.**
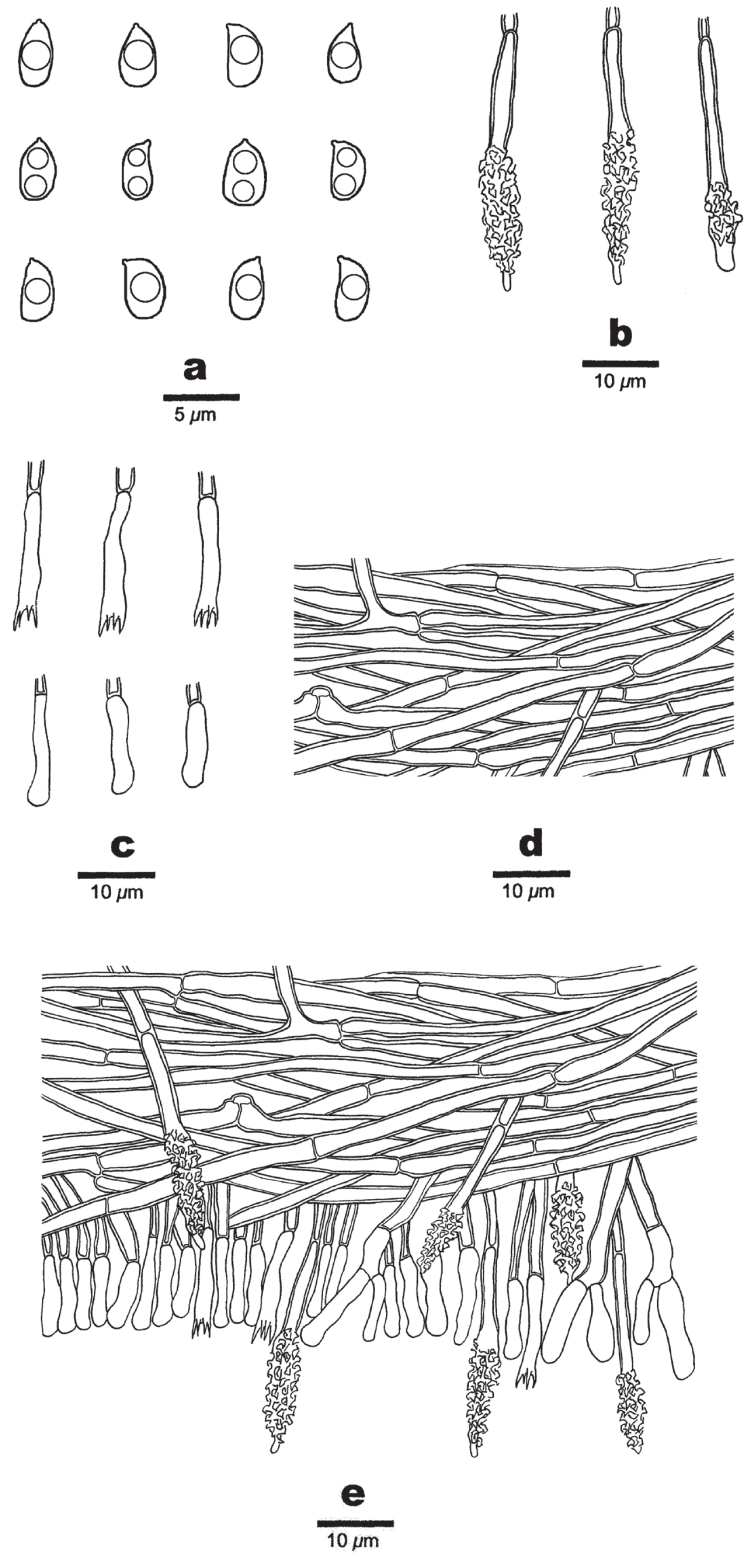
Microscopic structures of *Phanerochaetecastanea* (Holotype, Dai 24915) **a** basidiospores **b** cystidia **c** basidia and basidioles **d** vertical section of the subiculum **e** vertical section of the hymenium and subhymenium.

##### Description.

Basidiomata annual, resupinate, adnate, soft coriaceous, without odor and taste when fresh, detachable from substrate, up to 2 cm long, 1.5 cm wide, 100–200 µm thick. Hymenial surface smooth, whitish to yellowish when fresh, yellowish brown upon drying, becoming reddish brown in KOH. Sterile margins paler than hymenial surface, thinning out, usually with whitish rhizomorphs, up to 0.2 cm.

Hyphal system monomitic, generative hyphae mostly simple septate, occasionally with clamp connections, IKI–, CB–; tissue unchanged in KOH.

Subicular hyphae hyaline, thick-walled, up to 0.1 µm thick, simple septate, occasionally bearing clamp connections, occasionally branched, parallel to interwoven, 3–5 µm in diameter. Subhymenial hyphae hyaline, thick-walled, clampless, 1.5–3 µm in diameter.

Cystidia mostly subulate with a blunt or acute apex, 45–60 × 6–8 µm, hyaline, thick-walled, up to 0.1 µm thick, with a simple septum at the base, mostly encrusted with crystal granules at apical part, some with smooth apex, projecting up to 15 µm above the hymenial layer; cystidioles absent. Basidia clavate, with four sterigmata and a basal simple septum, 19–27 × 3–5.5 µm; basidioles of similar shape to basidia, but smaller.

Basidiospores ellipsoid, hyaline, thin-walled, smooth, usually with one or two medium guttules, IKI–, CB–, (3.7–)3.9–5.1(–5.2) × (2.2–)2.3–3.1 µm, L = 4.45 μm, W = 2.66 μm, Q = 1.67–1.68 (n=60/2).

##### Type of rot.

White rot.

##### Additional specimen examined (paratype).

China • Zhejiang Province, Jinhua, Wuyi County, Niutoushan Forest Park; on a rotten angiosperm wood; 17.VI.2023; Y.C. Dai 24916 (BJFC042469).

#### ﻿ Phanerochaete citrinoalba

Taxon classificationFungiPolyporalesPhanerochaetaceae

K.Y. Luo, Yuan Yuan, Y.C. Dai & Xin Zhang sp. nov.

8FC94ED8-1F57-5518-88C1-9D2D7CEB83CE

854772

Figs 4 , 5

##### Holotype.

China • Xizang Autonomous Region, Nyingchi, Sejila Mountain; on dead bamboo; 23.X.2023; Dai 26584 (BJFC044134).

**Figure 4.**
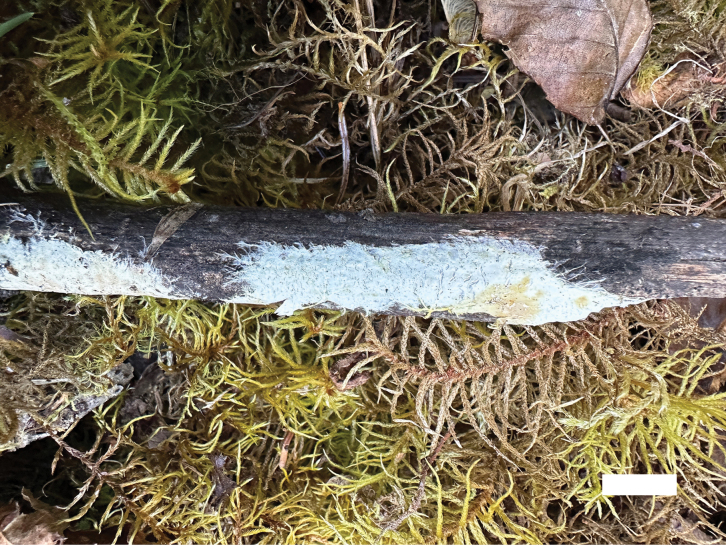
Basidiomata of *Phanerochaetecitrinoalba* (Holotype, Dai 26584). Scale bar: 1 cm.

##### Etymology.

*Citrinoalba* (Lat.) refers to the species having yellowish to whitish rhizomorphs.

**Figure 5.**
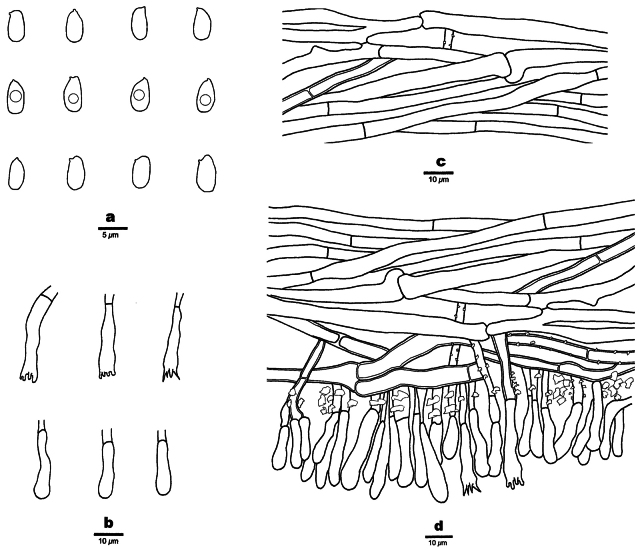
Microscopic structures of *Phanerochaetecitrinoalba* (Holotype, Dai 26584) **a** basidiospores **b** basidia and basidioles **c** vertical section of the subiculum **d** vertical section of the hymenium and subhymenium.

##### Description.

Basidiomata annual, resupinate, adnate, coriaceous, without odour and taste when fresh, up to 9 cm long, 1.5 cm wide, 70–130 µm thick. Hymenial surfaces smooth, cracking, white to cream when fresh, cream to slightly buff upon drying. Becoming greyish brown in KOH. Sterile margins distinct, concolorous with hymenial surface, usually with yellowish to whitish rhizomorphas, and up to 5 mm.

Hyphal system monomitic, generative hyphae mostly with simple septa, occasionally with clamp connections in subiculum, IKI–, CB–; tissue unchanged in KOH.

Subicular hyphae hyaline, thin- to thick-walled, frequently simple septate, occasionally bearing clamp connections, branched at acute angles, subparallel to interwoven, 3–6 µm in diameter. Subhymenial hyphae hyaline, thin- to thick-walled, clampless, 2–3.5 µm in diameter, encrusted with crystal granules.

Cystidia and cystidioles absent. Subhymenium frequently with crystal granules. Basidia clavate, with four sterigmata and a basal simple septum, 17–25 × 4–7 µm; basidioles of similar shape to basidia, but smaller.

Basidiospores ellipsoid to oblong ellipsoid, hyaline, thin-walled, smooth, some with a medium guttule, IKI–, CB–, (4.7–)4.9–6.3(–6.4) × (2.2–)2.4–3(–3.1) µm, L = 5.49 μm, W = 2.66 μm, Q = 2.06 (n = 30/1).

##### Type of rot.

White rot.

#### ﻿ Phanerochaete citrinorhizomorpha

Taxon classificationFungiPolyporalesPhanerochaetaceae

K.Y. Luo, Yuan Yuan, Y.C. Dai & Xin Zhang sp. nov.

85164717-2AD8-59C5-89FF-6915B5095550

854773

Figs 6 , 7

##### Holotype.

China • Zhejiang Province, Jinhua, Wuyi County, Xinzhai, Daozhi Village; on dead bamboo; 14.X.2023; Y.C. Dai 26417 (BJFC043967).

**Figure 6.**
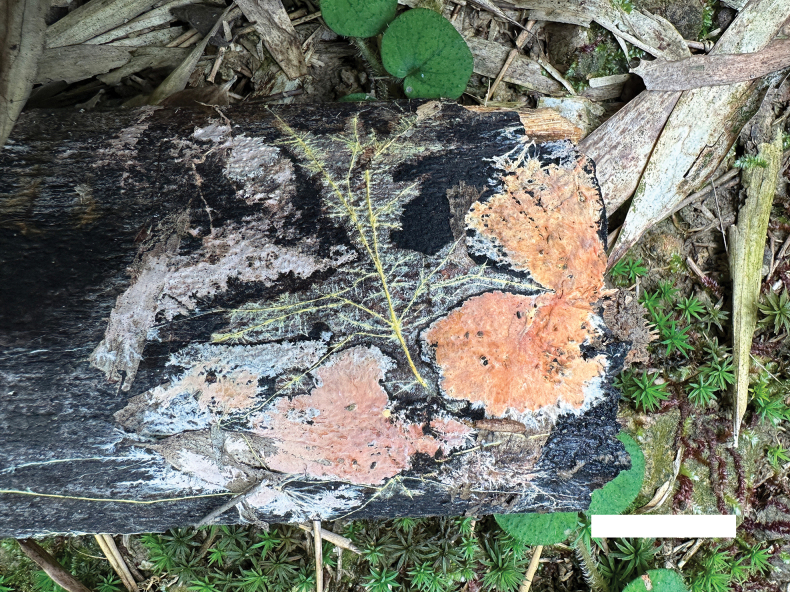
Basidiomata of *Phanerochaetecitrinorhizomorpha* (Holotype, Dai 26417). Scale bar: 2 cm.

##### Etymology.

*Citrinorhizomorpha* (Lat.) refers to the new species having yellowish rhizomorphs.

**Figure 7.**
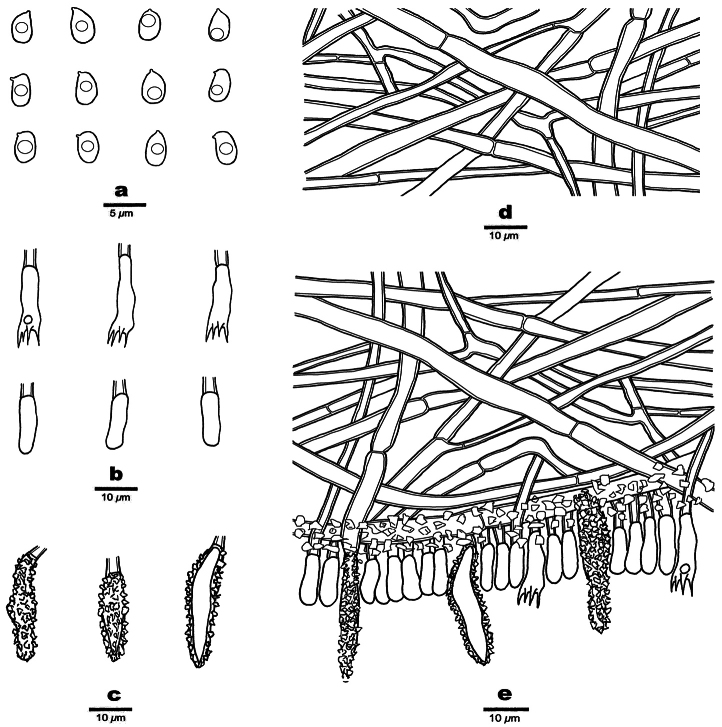
Microscopic structures of *Phanerochaetecitrinorhizomorpha* (Holotype, Dai 26417) **a** basidiospores **b** basidia and basidioles **c** cystidia **d** vertical section of the subiculum **e** vertical section of the hymenium and subhymenium.

##### Description.

Basidiomata annual, resupinate, adnate, soft coriaceous, without odor and taste when fresh, up to 7 cm long, 5 cm wide, 100–200 µm thick. Hymenial surfaces flesh-pink when juvenile, salmon to peach with age. Becoming purple in KOH. Sterile margins paler than hymenial surface, thinning out, usually with yellowish rhizomorphs, and up to 4 cm.

Hyphal system monomitic, generative hyphae simple septate, IKI–, CB–; tissue unchanged in KOH.

Subicular hyphae hyaline, thick-walled, up to 0.1 µm thick, simple septate, frequently branched at acute angles, interwoven, 2–6 µm in diameter. Subhymenial hyphae hyaline, thick-walled, 1.5–3 µm in diameter, encrusted with crystal granules.

Cystidia mostly subulate with an obtuse apex, hyaline, thick-walled, up to 0.1 µm thick, with a simple septum at the base, usually encrusted with crystal granules, projecting above hymenium, projecting up to 17 µm above the hymenial layer, 18–36 × 4–6 µm; cystidioles absent. Basidia clavate, with four sterigmata and a basal simple septum, 12–17 × 4–6 µm; basidioles of similar shape to basidia, but smaller.

Basidiospores ellipsoid, hyaline, thin-walled, smooth, usually with a medium guttule, IKI–, CB–, (3.3–)3.5–4.5(–4.9) × (1.8–)1.9–2.8(–3.2) µm, L = 3.94 μm, W = 2.37 μm, Q = 1.54–1.80 (n = 60/2).

##### Type of rot.

White rot.

##### Additional specimen examined (paratype).

China • Yunnan Province, Honghe, Jinping County, Fenshuiling Nature Reserve; on a fallen angiosperm branch; 18.VIII.2019; Y.C. Dai 20753 (BJFC032420).

#### ﻿ Phanerochaete wuyiensis

Taxon classificationFungiPolyporalesPhanerochaetaceae

K.Y. Luo, Yuan Yuan, Y.C. Dai & Xin Zhang sp. nov.

01418918-C9AB-5905-AADB-7C1CCC4B2850

854774

Figs 8 , 9

##### Holotype.

China • Zhejiang Province, Jinhua, Wuyi County, Shiehu Scenic Spot; on a fallen branch of *Pinusmassoniana*; 11.VIII.2023; Y.C. Dai 25530 (BJFC043078).

**Figure 8.**
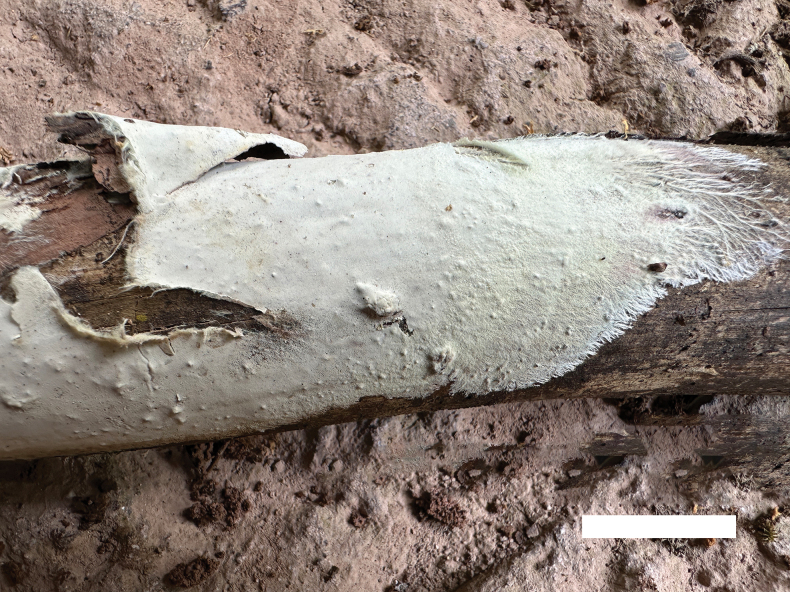
A basidioma of *Phanerochaetewuyiensis* (Holotype, Dai 25530). Scale bar: 2 cm.

##### Etymology.

*Wuyiensis* (Lat.) refers to “Wuyi County, Zhejiang Province, East China,” where the holotype was found.

**Figure 9.**
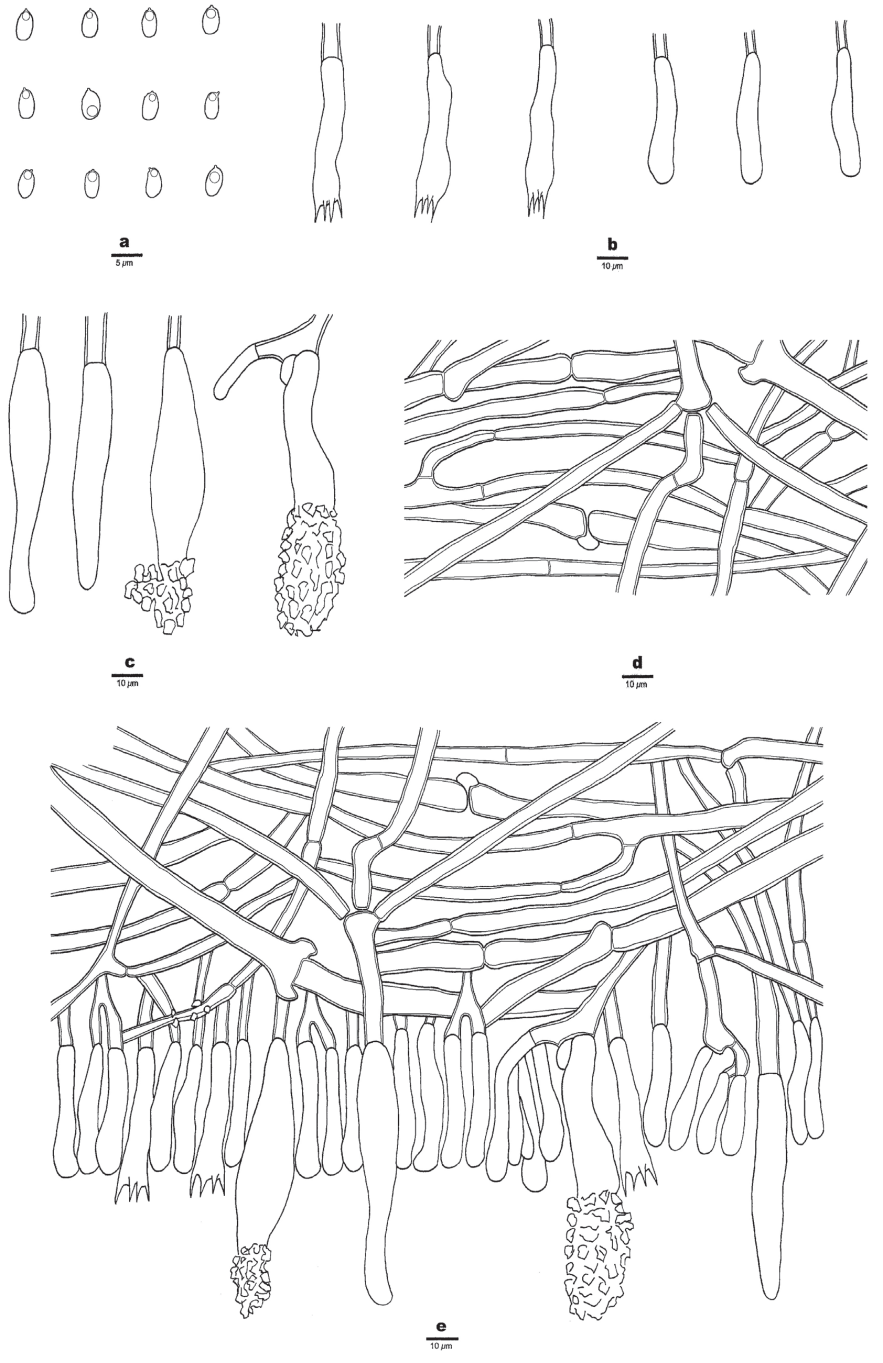
Microscopic structures of *Phanerochaetewuyiensis* (Holotype, Dai 25530) **a** basidiospores **b** basidia and basidioles **c** cystidia **d** vertical section of the subiculum **e** vertical section of the hymenium and subhymenium.

##### Description.

Basidiomata annual, resupinate, adnate, detachable from substrate, membranaceous, without odour and taste when fresh, up to 8 cm long, 3 cm wide, 200–300 µm thick. Hymenial surfaces smooth or locally tuberculate, uncracked, whitish when fresh and upon drying. Becoming lemon-yellow in KOH. Sterile margins distinct, concolorous with hymenial surface, with whitish rhizomorphs, and up to 1 cm.

Hyphal system monomitic, generative hyphae with simple septa and clamp connections, IKI–, CB–; tissue unchanged in KOH.

Subicular generative hyphae hyaline, thick-walled with simple septa and clamp connections, usually constricted at simple septa, sometimes with three branches at a single septum, interwoven, 3–16 µm in diameter. Subhymenial hyphae hyaline, thick-walled clampless, branched present, 2–3.5 µm in diameter.

Cystidia clavate to fusiform, hyaline, thin-walled, with a simple septum at the base, some apically encrusted with crystal granules, projecting above hymenium, 22–44 × 6–10 µm; cystidioles absent. Basidia clavate, with four sterigmata and a basal simple septum, 13–25 × 4–7 µm; basidioles of similar shape to basidia, but smaller.

Basidiospores ellipsoid, hyaline, thin-walled, smooth, with one or two medium guttules, IKI–, CB–, (3.3–)3.6–4.6(–5.4) × (1.8–)2.1–3.2(–3.4) µm, L = 4.07 μm, W = 2.48 μm, Q = 1.61–1.68 (n = 90/3).

##### Type of rot.

White rot.

##### Additional specimens examined (paratype).

China • Zhejiang Province, Wuyi County, Shiehu Scenic Spot; on a fallen branch of *Pinusmassoniana*; 12.X.2023; Y.C. Dai 26246 (BJFC043796) • *ibid.* on rotten wood of *Pinusmassoniana*; 12.X.2023; Y.C. Dai 26250 (BJFC043800).

## ﻿Discussion

Taxa of the genus *Phanerochaete* are important components of woody plant ecosystems, and they have the ability to decompose rotten wood in forest or bamboo ecosystems. Many species in the genus have been described from the subtropics and tropics in recent years (Chen et al. 2021; Li et al. 2023; Yu et al. 2023; Zhang et al. 2023; Deng et al. 2024). In the present study, four new species, *viz. Phanerochaetecastanea*, *P.citrinoalba*, *P.citrinorhizomorpha* and *P.wuyiensis*, are described based on a combination of morphological features and molecular evidence.

Phylogenetically, based on ITS+nLSU topology (Fig. 1), four new species were nested in the *Phanerochaete* clade. Among them, *P.castanea* grouped together with *P.metuloidea*, however, *P.metuloidea* is delimited from *P.castanea* by having greyish orange, brownish orange to light brown hymenophore and longer basidia (40–70 × 5–8.5 μm vs. 19–27 × 3–5.5 µm, Xu et al. 2020). *Phanerochaetecitrinoalba* was sister to *P.burtii*, but *P.burtii* differs from *P.citrinoalba* by having cylindric cystidia (Parmasto 1967). *P.citrinorhizomorpha* grouped together with *P.minor*, however, *P.minor* is different from *P.citrinorhizomorpha* by having membranaceous basidiomata and apically encrusted cystidia (Xu et al. 2020). *Phanerochaetewuyiensis* was sister to *P.subtropica*, but *P.subtropica* differs from *P.wuyiensis* by its coriaceous basidiomata (Yu et al. 2023).

Morphologically, *Phanerochaetecastanea* resembles *P.burdsallii* Y.L. Xu et al., *P.hymenochaetoides* Y.L. Xu & S.H. He and *P.laevis* (Fr.) J. Erikss. & Ryvarden by sharing hymenophore becoming reddish brown or red in KOH. However, *P.burdsallii* is different from *P.castanea* by its membranaceous basidiomata and longer basidiospores (5.3–6 × 2.5–3 μm vs. 3.9–5.1 × 2.3–3.1 µm, Xu et al. 2020); *P.hymenochaetoides* is distinguished from *P.castanea* by its basidiomata without rhizomorphs and subicular hyphae encrusted with yellow resinous granules (Xu et al. 2020); *P.laevis* is different from *P.castanea* by its membranaceous basidiomata, ochraceous to cinnamon hymenial surface, and longer basidia (30–50 × 4–5 μm vs. 19–27 × 3–5.5 µm, Eriksson et al. 1978).

*Phanerochaetecitrinoalba* resembles *P.daliensis* J. Yu & C.L. Zhao, *P.subtuberculata* J. Yu & C.L. Zhao and *P.tongbiguanensis* Y.L. Deng & C.L. Zhao by sharing a coriaceous basidiomata. However, *P.daliensis* is different from *P.citrinoalba* by its grandinioid hymenial surface and thick-walled basidiospores (Yu et al. 2023); *P.subtuberculata* is distinguished from *P.citrinoalba* by having tuberculate hymenial surface, generative hyphae with simple septa, and clavate cystidia (Yu et al. 2023); and *P.tongbiguanensis* is different from *P.citrinoalba* by its generative hyphae with simple septa, subclavate cystidia and bigger basidiospores (6–9 × 3–4.5 μm vs. 4.9–6.3 × 2.4–3 µm, Deng et al. 2024).

*Phanerochaetecitrinorhizomorpha* is similar to *P.cinerea* Y.L. Xu & S.H. He, *P.guangdongensis* C.C. Chen et al., and *P.spadicea* C.C. Chen & Sheng H. Wu by sharing generative hyphae with simple septa. However, *P.cinerea* is distinguished from *P.citrinorhizomorpha* by having grey, brownish grey to greyish brown hymenophore, the absence of cystidia, and longer basidiospores (4.8–5.6 × 2–2.5 μm vs. 3.5–4.5 × 1.9–2.8 µm, Xu et al. 2020); *P.guangdongensis* is different from *P.citrinorhizomorpha* by its membranaceous to subceraceous basidiomata, buff to yellowish brown hymenial surface, and longer basidiospores (6.9–7.8 × 2.6–3 μm vs. 3.5–4.5 × 1.9–2.8 µm, Chen et al. 2021); *P.spadicea* is distinguished from *P.citrinorhizomorpha* by having membranaceous basidiomata, buff to pale brown hymenial surface, and longer basidiospores (4.5–5 × 1.9–2.2 μm vs. 3.5–4.5 × 1.9–2.8 µm, Chen et al. 2021).

*Phanerochaetewuyiensis* is similar to *P.burdsallii* Y.L. Xu et al., *P.crystallina* C.C. Chen et al., and *P.subrosea* Y.L. Xu & S.H. He by sharing membranaceous basidiomata. However, *P.burdsallii* is different from *P.wuyiensis* by its hymenophore becoming reddish brown in KOH and longer basidiospores (5.3–6 × 2.5–3 µm vs. 3.6–4.6 × 2.1–3.2 µm, Xu et al. 2020); *P.crystallina* is different from *P.wuyiensis* by having cream to ochraceous-buff hymenial surface and longer basidiospores (5.1–5.7 × 2.2–2.5 µm vs. 3.6–4.6 × 2.1–3.2 µm, Chen et al. 2021); and *P.subrosea* is readily distinguished from *P.wuyiensis* by its hymenophore turning purple in KOH and longer basidiospores (5–6 × 2.5–3 µm vs. 3.6–4.6 × 2.1–3.2 µm, Xu et al. 2020).

Xizang Autonomous Region, Yunnan Province in southwest China, and Zhejiang Province in eastern China are very rich for wood-inhabiting fungi. Numerous taxa of such fungi have been described from these areas based on morphological and molecular phylogenetic analyses (Volobuev et al. 2015; Chen et al. 2018; Ordynets et al. 2018; Cui et al. 2019; Dai et al. 2021; Wu et al. 2022a, 2022b; Zhang et al. 2023; Zhou et al. 2023; Zhao et al. 2024). DNA sequence data are very useful in exploring cryptic taxa and the diversity of corticioid fungi. In the present study, four new species of *Phanerochaete* are described from these two areas, which improve our knowledge of the diversity of Chinese white rot fungi.

### ﻿Key to the accepted species of *Phanerochaete* in China

**Table:** 

1	Hymenophore poroid	***P.inflata***
–	Hymenophore non-poroid	**2**
2	Hymenophore grandinioid	**3**
–	Hymenophore smooth to raduloid	**5**
3	Basidiospores thick-walled	***P.daliensis***
–	Basidiospores thin-walled	**4**
4	Cystidia present	***P.aculeata***
–	Cystidia absent	***P.yunnanensis***
5	Hymenophore at first smooth, odontioid to raduloid when mature	**6**
–	Hymenophore smooth, more or less tuberculate	**7**
6	Distributed in northern China	***P.cumulodentata***
–	Distributed in southern China	***P.magnoliae***
7	Rhizomorpha present	**8**
–	Rhizomorpha absent	**19**
8	Hymenophore purple in KOH	**9**
–	Hymenophore unchanged, or becoming buff, grayish brown, brown, reddish brown, red or black in KOH	**10**
9	Cystidia without crystal granules	***P.subrosea***
–	Cystidia with crystal granules	***P.citrinorhizomorpha***
10	Hyphal cords reddish brown	***P.citrinosanguinea***
–	Hyphal cords white, cream, grayish or orange	**11**
11	Cystidia absent	***P.citrinoalba***
–	Cystidia present	**12**
12	Cystidia obviously encrusted with crystals	***P.laevis***
–	Cystidia smooth or sparsely encrusted	**13**
13	Generative hyphae without clamp connections in subiculum	***P.subsanguinea***
–	Generative hyphae with clamp connections in subiculum	**14**
14	Cystidia thick-walled	**15**
–	Cystidia thin-walled	**16**
15	Cystidia with septa	***P.subtropica***
–	Cystidia without septa	***P.castanea***
16	Hyphal cords turning reddish brown in KOH	**17**
–	Hyphal cords not turning reddish brown in KOH	**18**
17	Cystidia 30–70 × 4–6 µm; basidiospores 5–6 × 2.5–3 µm	***P.leptocystidiata***
–	Cystidia 35–50 × 4–6 µm; basidiospores 4–5 × 2–2.5 µm	***P.sinensis***
18	Basidiomata buff in KOH	***P.shenghuaii***
–	Basidiomata darkening in KOH	***P.rhizomorpha***
19	Cystidia absent	**20**
–	Cystidia present	**24**
20	Hymenophore brown	***P.porostereoides***
–	Hymenophore whitish, cream, gray, grayish brown, or yellowish	**21**
21	Hymenial surface lightly darkening in KOH	**22**
–	Hymenial surface unchanged in KOH	**23**
22	Basidiomata undetachable from substrate	***P.pruinosa***
–	Basidiomata detachable from substrate	***P.cinerea***
23	Basidiospores 4.2–5.2 × 1.8–2.2 µm	***P.spadicea***
–	Basidiospores 4.2–5.1 × 2.5–3.3 µm	***P.brunnea***
24	Cystidia obviously encrusted	**25**
–	Cystidia smooth or sparsely encrusted	**36**
25	Cystidia encrusted with yellow resinous granules	**26**
–	Cystidia encrusted without yellow resinous granules	**27**
26	Hymenophore brown; quasi-binding hyphae present	***P.ericina***
–	Hymenophore lilac pink; quasi-binding hyphae absent	***P.incarnata***
27	On Monocotyledons	***P.minor***
–	On Dicotyledons	**28**
28	Cystidia up to 150 µm long	***P.velutina***
–	Cystidia up to 80 µm long	**29**
29	Cystidia up to 13 µm wide	**30**
–	Cystidia up to 9 µm wide	**31**
30	Cystidia only apically encrusted; widely distributed in China	***P.concrescens***
–	Cystidia encrusted up to one third of the length; distributed only in southern China	***P.australis***
31	Hymenophore yellow to buff	**32**
–	Hymenophore white to cream	**34**
32	Basidiomata ceraceous; basidiospores > 5.5 µm long	***P.livescens***
–	Basidiomata membranaceous; basidiospores < 5.5 µm long	**33**
33	Hymenophore yellow to yellowish brown; margin determinate	***P.hymenochaetoides***
–	Hymenophore cream to light yellow; margin fibrillose	***P.cystidiata***
34	Cystidia thick-walled; basidia up to 70 µm long, 8.5 µm wide	***P.metuloidea***
–	Cystidia thin- to slightly thick-walled; basidia up to 50 µm long, 6 µm wide	**35**
35	Subicular hyphae thin to slightly thick-walled; cystidia subulate	***P.laevis***
–	Subicular hyphae thick-walled; cystidia tapering but with obtuse apex	***P.sordida***
36	Cystidia two kinds	***P.robusta***
–	Cystidia one kind	**37**
37	Clamp connections present in subiculum	**38**
–	Clamp connections absent in subiculum	**43**
38	Basidiospores < 4.6 µm in length	**39**
–	Basidiospores > 4.6 µm in length	**41**
39	Cystidia < 40 µm in length	***P.albida***
–	Cystidia > 40 µm in length	**40**
40	Basidiomata turning lemon-yellow in KOH	***P.wuyiensis***
–	Basidiomata turning greyish green in KOH	***P.carnosa***
41	Cystidia < 40 µm in length	***P.subcarnosa***
–	Cystidia > 40 µm in length	**42**
42	Basidiomata margin fibrillose	***P.affinis***
–	Basidiomata margin byssoid	***P.alpina***
43	On bamboo	**44**
–	On wood	**45**
44	Basidiospores > 6.2 µm in length	***P.bambucicola***
–	Basidiospores < 6.2 µm in length	***P.parmastoi***
45	Basidiospores cylindrical	**46**
–	Basidiospores ellipsoid	**48**
46	Basidiospores > 6 µm in length	***P.guangdongensis***
–	Basidiospores < 6 µm in length	**47**
47	Basidiomata coriaceous	***P.subtuberculata***
–	Basidiomata membranaceous	***P.crystallina***
48	Basidiomata coriaceous to soft corky	***P.hainanensis***
–	Basidiomata membranaceous	**49**
49	Basidiospores < 6.5 µm in length	***P.tongbiguanensis***
–	Basidiospores > 6.5 µm in length	**50**
50	Basidiospores with oil-drops	***P.taiwaniana***
–	Basidiospores without oil-drops	***P.stereoides***

## Supplementary Material

XML Treatment for Phanerochaete castanea

XML Treatment for Phanerochaete citrinoalba

XML Treatment for Phanerochaete citrinorhizomorpha

XML Treatment for Phanerochaete wuyiensis

## ﻿Additional information

### Author ORCIDs

Kai-Yue Luo https://orcid.org/0000-0001-6145-4864

Xin Zhang https://orcid.org/0009-0005-8363-7852

Yu-Cheng Dai https://orcid.org/0000-0002-6523-0320

Yuan Yuan https://orcid.org/0000-0001-6674-9848

### Data availability

All of the data that support the findings of this study are available in the main text.
